# Opportunistic Tool Use by Two Unexpected Corvid Species

**DOI:** 10.1002/ece3.71314

**Published:** 2025-05-11

**Authors:** Tanita Giri, Elias Garcia‐Pelegrin

**Affiliations:** ^1^ Department of Psychology National University of Singapore Singapore Singapore

**Keywords:** avian cognition, Corvidae, corvids, evolution, house crow, Sunda crow, tool use

## Abstract

This Nature note reports the first documented instance of tool use in Sunda crows (*Corvus enca*) and provides additional evidence of this ability in house crows (*Corvus splendens*). At Singapore Zoo (December 2023), individuals from both species spontaneously manipulated a hooked stick to extract food rewards from enclosed containers. This observation extends the catalogue of tool‐using corvids. We briefly review tool use across the corvid family and examine competing hypotheses regarding its evolution, including inherited predisposition from a tool‐using ancestor and the development of general physical intelligence with food caching as a potential precursor. Our findings suggest that the cognitive foundation for tool use may be conserved across the corvid family, with expression contingent upon environmental demands rather than species‐specific adaptations. This work contributes to ongoing discussions regarding the evolutionary origins of complex problem‐solving in birds and the potential role of general physical intelligence in corvid cognition.

## Graphical abstract

Picture exemplifying the crow tool use.

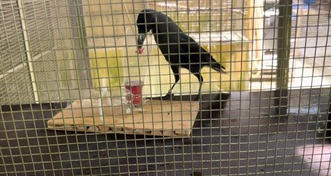

1

Several corvid species demonstrate various forms of tool use, either in their natural habitats or in controlled laboratory environments. The wide range of genetically related species exhibiting such complex behaviour allows for hypotheses regarding its evolution and the cognitive abilities of their common ancestor. Although there's much debate on the precise definition of tool use, most experts agree it involves using a manipulable object to change another object's physical characteristics through mechanical interactions (Shumaker et al. 2011). This current report details an event where a house crow ( *Corvus splendens* ) and a Sunda crow ( *Corvus enca* ) entered the research enclosure and spontaneously solved unattended tool‐use tasks. House crows have previously been reported to use tools in captivity (Rajan and Balasubramanian 1989), but this is the first documentation of Sunda crows using tools. We first describe the instance of tool use before briefly exploring what this event suggests about the potential evolution of tool use in this cognitively advanced family of birds.

Direct observations were made on the 17/12/2023, at Singapore Zoo (Mandai Wildlife Reserve). At the research facilities, a group of three male house crows and two male Sunda crows, rescued after being found unable to survive in the wild a year before the observations were made, have free access to the enclosures of Asian hornbills and parrots. While researchers were testing physical intelligence with a tool‐use device, the crows entered and interacted with it. The apparatus, inspired by the Bird and Emery (2009) study, was constructed using two cleaned and cut recycled plastic bottles, shaped into long tubes and secured to an acrylic board with zip ties (Figure 1). Each tube measured 15 cm in height and 6 cm in circumference. The task required inserting a hook tool to retrieve a cup with a handle filled with protein pellets from the bottle. The tool itself was a chopstick with a 3D printed hook attached to each end.

**FIGURE 1:**
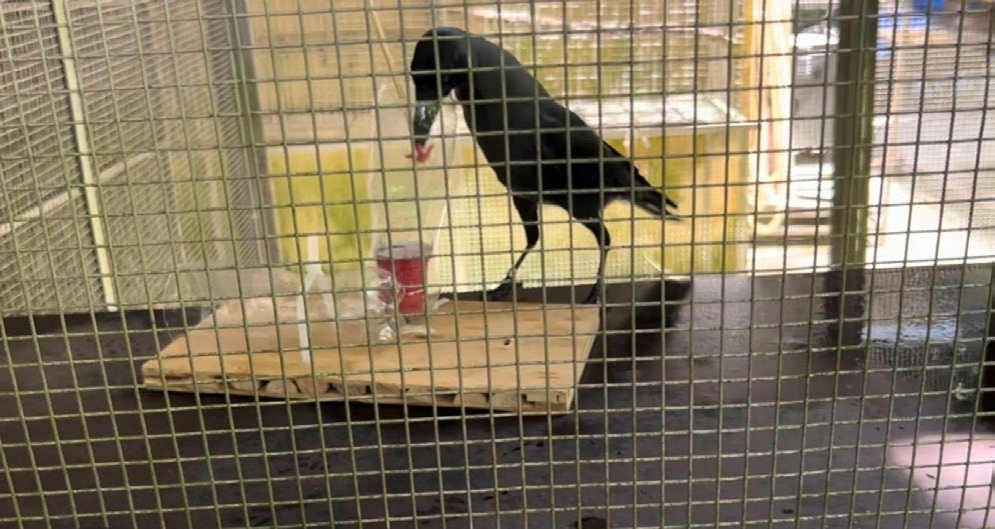
Picture of tool use apparatus and house crow ( *Corvus splendens* ) inserting hooked tool.

The first observed interaction occurred when a Sunda crow flew into the hornbill enclosure, picked up the stick, and carried it to a nearby perch. The crow manipulated the tool, attempting to cache it in a crevice, while other Sunda and house crows pecked at the soda bottles, unsuccessfully trying to access the pellets inside. The focal Sunda crow then returned to the apparatus and, after several attempts, managed to insert the stick into the bottle and retrieve the cup containing the food rewards. This successful action triggered fierce competition among the crows for the pellets. To document the tool use, the apparatus was re‐baited and filmed. This time, a house crow took the hooked stick, examined the bottle, inserted the tool, and retrieved the reward (see Video 1). Notably, at the time of the experiment, the hornbills had not interacted with the tools, meaning the crows had no prior observational experience of the task being solved. The Sunda crow's tool use was a spontaneous innovation, and after a single observation, the house crow successfully solved the task, likely at least partially through social learning. Beyond this, the crows had no additional experience with the materials or task. Testing was then halted to prevent the hornbill and parrot subjects from gaining observational experience from watching the crows.

**VIDEO 1:** Recorded interaction of house crow (*Corvus splendens*) solving the tool use apparatus. Video content can be viewed at https://onlinelibrary.wiley.com/doi/10.1002/ece3.71314

The Indian house crow ( *Corvus splendens* ), indigenous to the Indian subcontinent, is an omnivorous, aggressive, and opportunistic scavenger (Ali 2008). It is classified as an invasive species that has dispersed across East and Southeast Asia, Africa, the Middle East, and various oceanic islands (Brook et al. 2003). The extensive spread of house crows can be attributed to both deliberate introductions and maritime migrations facilitated by increased global sea traffic (Long 1981). This species exhibits remarkable adaptability to urban environments, where it forages on anthropogenic food sources, human refuse, animal carcasses, invertebrates, and juvenile birds (Feare and Mungroo 1990). Indeed, their success in urban settings has been largely ascribed to their preference for consuming human‐related food waste rather than natural plant or animal matter (Kumar and Ojha 2023). House crows typically forage in groups, often alongside other urban bird species, during the early morning and late evening hours (Anjum et al. 2021). They are monogamous and breed year‐round, with a higher frequency of breeding during the hotter and drier seasons (Akter et al. 1994; Nordin and Yusof 1980).

The Sunda crow ( *Corvus enca* ), previously referred to as the slender‐billed crow, is distributed across Indonesia and Peninsular Malaysia (Clements et al. 2023). It predominantly inhabits moist lowland forests and is typically found near watercourses, clearings, or the edges of forests (Eddy et al. 2021). Given that the Sunda crow was only recently differentiated from the slender‐billed crow, limited information is available regarding its ecology.

Flexible tool use relies on the dynamic interactions with objects and is often developed through learning experiences. These interactions can greatly differ among individuals and species and are often believed to require advanced cognitive abilities, such as sensorimotor learning and coordination, causal reasoning, extensive working memory, and a functional understanding of objects (Hunt et al. 2013). While various birds and mammals show flexible tool use (see Shumaker et al. 2011 for a review), the corvid family stands out for the high number of species that have been reported to use tools. In our discussions, we will focus on instances of true foraging‐related flexible true tool use in corvids, which involves items such as hammers and probes that are detached from the substrate, being held directly in the beak or foot (Boswall 1977). It should be noted that some species of corvids have been reported to make use of tools to conceal caches in the wild (Heinrich 1999). This instance of tool use seems to serve as a response to competitor threat rather than to aid in foraging; hence, for the purpose of this discussion, we will not consider this as an instance of foraging‐related natural tool use. Following this, among this family, only New Caledonian crows ( *Corvus moneduloides* ) and Hawaiian crows ( *Corvus hawaiiensis* ) naturally use tools. This behavior is likely an adaptive response to ecological pressures such as the abundance of concealed food due to a lack of competitors skilled in extractive foraging (Kenward et al. 2004) and low predator threat (Kacelnik et al. 2006) among others. Ecological demands have led natural tool‐using crows to adapt morphologically for this task. Unlike other corvids with curved bills, these crow species exhibit relatively straight bills and large eyes with wide binocular overlap, aiding in precise tool handling (Troscianko et al. 2012). In contrast, other corvid species such as ravens, rooks, and jays, which are not natural tool users, have shown the ability to use tools to solve novel foraging problems in laboratory settings. This encompasses the creation and use of probe and hook tools, as well as the dropping of stones, all of which are intended to retrieve food items that would otherwise be inaccessible (refer to Table 1).

**TABLE 1:** Corvid species documented to employ tool use in wild and captive contexts.

Species	Binomial nomenclature	Type of tool use	Paper	Comments
New Caledonian Crows	*Corvus moneduloides*	Manufacture of hooked‐twig and stepped‐cut barbed pandanus leaf to capture prey	Hunt (1996)	NA
Common Ravens	*Corvus corax*	(1) Used a feather to reach into a crevice to obtain a food cache (2) Dropped a stone to collapse a platform (3) Hammered a snail with a stone	(1) Gallot & Gruber (2019) (2) Kabadayi & Osvath (2017) (3) Rezanov & Rezanov (2010) Extracted from Jacobs & Osvath (2023)	NA
Rooks	*Corvus frugilegus*	Dropped stones into vertical tubes to collapse platform and obtain reward Manufactured hook sticks to lift a bucket containing a reward from a vertical tube	Bird and Emery (2009)	They demonstrated the ability to select functional tools to solve problems
American crows	*Corvus brachyrhynchos*	(1) Used stones to smash acorn (2) Used cups to transport water to dry mash (3) Modified and used a piece of food as a probe, poking into tiny holes to retrieve spiders	(1*) Duvall in Boswall (1978) (2*) Beck (1980) (3) Caffrey (2000) *Extracted from Lefebvre et al. (2002)	NA
Hawaiian crows	*Corvus hawaiiensis*	Used sticks as probe tools	Rutz et al. (2016)	NA
House crows	*Corvus splendens*	Used leaf to get ants from hole	Rajan and Balasubramanian (1989) Extracted from Lefebvre et al. (2002)	NA
Northwestern crows	*Corvus caurinus*	Used stick to pry open peanut	Jewett in Boswall (1983) Extracted from Lefebvre et al. (2002)	NA
Fan‐tailed raven	*Corvus rhipidurus*	Hammered egg with rock	Andersson (1989) Extracted from Lefebvre et al. (2002)	NA
Eurasian jays	*Garrulus glandarius*	(1) Aesop's fable: dropped items into a cylinder containing water, in order to displace the water and retrieve a floating food reward (2) Dropped stones into vertical tubes to collapse platform and obtain reward	(1) Cheke et al. (2011) (2) Amodio et al. (2019)	NA
Northern blue jays	*Cyanocitta cristata*	Teared pieces of newspaper and used them to obtain out of reach food	Jones & Kamil (1973)	NA
Western scrub‐jays	*Aphelocoma californica*	Aesop's fable: dropped items into a cylinder containing water, in order to displace the water and retrieve a floating food reward	Logan et al. (2016)	They did not demonstrate an understanding of the functional difference between tools.

*Note:* We focused on extractive foraging tool‐use for the purpose of this report. There may be other instances of tool use in other corvid species that are not mentioned in this table.

Corvids, in general, exhibit more innovative foraging techniques compared to other bird species, indicating a greater behavioural adaptability (Rutz and St Clair 2012), and demonstrate an inherent understanding of tool functionality regardless of whether they are natural tool users or not (e.g., Bird and Emery 2009). Additionally, corvids possess an unusually enlarged caudolateral nidopallium (NCL), which functions similarly to the prefrontal cortex in mammals. This structure is crucial for cognitive abilities such as visual working memory, associative learning, and the abstraction of general principles, all of which may play a role in tool use (see Cabrera‐Álvarez and Clayton 2020). Therefore, it is highly probable that a general cognitive ability for physical intelligence existed prior to corvid speciation. In this scenario, the common ancestor needed a sophisticated form of physical intelligence to thrive in their ecosystem. Hence, all descendants inherited not the tool‐using behaviour itself but the cognitive foundation to understand cause‐and‐effect relationships that could lead to tool use when necessary (Kacelnik 2009). For example, rooks have been observed to manipulate bin liners at a motorway service station in England to acquire food from inside the bins, demonstrating an instance of opportunistic tool use by a non‐tool using corvid in complex, urban environments (Clayton and Emery 2005).

Particularly, food caching has been proposed as an evolutionary precursor to tool use, supporting the theory of ancestral general physical intelligence in corvids (Rutz and St Clair 2012). This connection is unsurprising, given the structural and developmental parallels between caching behaviours and tool use. Both involve the placement of objects into confined spaces with subsequent adjustments, often in the context of accessing or storing food. In caching, corvids hide food in secret locations, thereby gaining exclusive knowledge of its location—an advantage similar to how tool use enables access to concealed resources. Additionally, the handling of twigs during early development can serve as a precursor to both behaviours (Schloegl et al. 2011).

Initial manifestations of these behaviours are often repetitive and lack immediate function. For instance, New Caledonian crows exhibit back‐and‐forth movements with sticks in their beaks (proto‐probing), while ravens press inedible objects against surfaces (placement), suggesting that these complex behaviours may evolve from basic exploratory actions (Kenward et al. 2006; Bugnyar et al. 2007; Amodio et al. 2018). Thus, it is plausible that ancestral corvids' innate tendencies to manipulate objects facilitated the adaptation of caching mechanisms, ultimately giving rise to tool use in foraging among New Caledonian crows (Schloegl et al. 2011; Jacobs et al. 2014). Another possibility is that these evolutionary tendencies for tool use are exclusive to the genus *Corvus*, which explains why such behaviours are predominantly observed in crows, ravens, and rooks. This trait may have developed convergently in some other non‐*Corvus* corvids, who also seem to exhibit tool use under laboratory conditions—in a water displacement task and object dropping task to retrieve an otherwise inaccessible reward (see Cheke et al. 2011; Amodio et al. 2019).

Alternatively, all corvids may have an inherent capacity for tool use, with this behaviour emerging only in species facing relevant foraging challenges. The common ancestor of corvids, like the New Caledonian crow, may have occupied a niche where tool use was essential for obtaining food. As corvids underwent speciation and adapted to diverse ecological settings, they began exploiting food resources more efficiently served by other feeding techniques and morphological traits. Consequently, the ability to use tools, although seemingly inherent in corvid species, is expressed based on the ecological environment's demands. This hypothesis can account for why, in controlled settings where the environment pressures for tool use, these non‐habitual tool users quickly exhibit tool‐use behaviours. Particularly, in captivity, the absence of predators and simplified foraging due to human‐provided food might allow these birds to focus on problem‐solving tasks and explore tools, increasing the chances of developing tool‐use behaviours (Haslam 2013). However, the first hypothesis appears more likely, as Corvus has undergone niche expansions accompanied by phenotypic adaptations, such as the development of new beak morphologies (Garcia‐Porta et al. 2022).

In summary, tool use appears to be a common behavioural trait among corvids. This report confirms that the house crow can use tools and adds the Sunda crow to the growing list of corvid species able to use tools. Together, they suggest that corvids can employ tools for extractive foraging when required by their environment. Further studies on the tool‐using abilities of various corvid species, whether in natural settings or controlled experiments, their understanding of cause‐and‐effect relationships, and the physical characteristics of the tools utilized, will provide additional insights. This expanding body of research promises to unlock the evolutionary origins of this remarkable behaviour within the corvid family, often regarded as “feathered apes” (Emery 2004).

## Author Contributions

**Tanita Giri:** writing – original draft (equal). **Elias Garcia‐Pelegrin:** conceptualization (lead), data curation (lead), investigation (lead), writing – original draft (equal), writing – review and editing (lead).

## Conflicts of Interest

The authors declare no conflicts of interest.

## Supporting information


Data S1.

## Data Availability

All data supporting the findings reported in this study are included in the manuscript.
