# Supplementary material for: Opportunistic Tool Use by Two Unexpected Corvid Species
Source: Ecol Evol. 2025 May 11;15(5):e71314. doi: 10.1002/ece3.71314 (PMC12066819; doi:10.1002/ece3.71314)
Supplement: Supplementary file 1 — Data S1. [file ECE3-15-e71314-s001.docx]

**References for Table 1**

Amodio, P., Boeckle, M., Jelbert, S. A., Ostoijc, L., & Clayton, N. S. (2019). How flexible is tool use in

Eurasian jays (Garrulus glandarius)?. *bioRxiv*, 803700.

Andersson, S. (1989). Tool Use by the Fan-Tailed Raven (Corvu srhipidurus). *The Condor*, *91*(4), 999-999.

Beck, B. B. (1980). Animal tool behavior: The use and manufacture of tools by animals. *(No Title)*.

Bird, C. D., & Emery, N. J. (2009). Insightful problem solving and creative tool modification by captive nontool-using rooks. *Proceedings of the National Academy of Sciences*, *106*(25), 10370-10375.

Caffrey, C. (2000). Tool modification and use by an American Crow. *The Wilson Bulletin*, *112*(2), 283-284.

Cheke, L. G., Bird, C. D., & Clayton, N. S. (2011). Tool-use and instrumental learning in the Eurasian jay (Garrulus glandarius). *Animal Cognition*, *14*, 441-455.

Boswall, J. (1978) Further notes on tool-using in birds and related behaviour.*Aviculture Magazine*, *84*, 162-166.

Boswall, J. (1983). Tool-using and related behaviour in birds: more notes. *Aviculture Magazine*, *89*, 94-108.

Gallot, Q., & Gruber, T. (2019). Spontaneous use and modification of a feather as a tool in a captive common raven. *Ethology*, 125, 755–758. <https://doi.org/10.1111/eth.12928>

Hunt, G. R. (1996). Manufacture and use of hook-tools by New Caledonian crows. *Nature*, *379*(6562), 249-251.

Jacobs, I., & Osvath, M. (2023). Tool use and tooling in ravens (Corvus corax): A review and novel observations. Ethology, 129(3), 169-181.

Jones, T. B., & Kamil, A. C. (1973). Tool-making and tool-using in the northern blue jay. *Science*, *180*(4090), 1076-1078.

Kabadayi, C., & Osvath, M. (2017). Ravens parallel great apes in flexible planning for tool-use and bartering. *Science*, 357, 202–204. <https://doi.org/10.1126/science.aam8138>

Lefebvre, L., Nicolakakis, N., & Boire, D. (2002). Tools and brains in birds. *Behaviour*, *139*(7), 939-973.

Logan, C. J., Harvey, B. D., Schlinger, B. A., & Rensel, M. (2016). Western scrub-jays do not appear to attend to functionality in Aesop’s Fable experiments. *PeerJ*, *4*, e1707.\

Rajan, S. A., & Balasubramanian, P. (1989). Toolusing behaviour in Indian House Crow Corvus splendens. *J. Bombay Nat. Hist. Soc*, *86*, 450.

Rezanov, A. A., & Rezanov, A. G. (2010). Ворон *Corvus corax* разбивает улитку с помощью камня. *Русский орнитологический журнал*, 19, 1478–1480.

Rutz, C., Klump, B. C., Komarczyk, L., Leighton, R., Kramer, J., Wischnewski, S., Sugasawa, S., Morrissey, M. B., James, R., St Clair, J. J. H., Switzer, R. A., & Masuda, B. M. (2016). Discovery of species-wide tool use in the Hawaiian crow. *Nature*, *537*(7620), 403-407.
